# Supplementary material for: Development and initial validation of a simple tool to screen for partner support or opposition to HIV prevention product use
Source: PLoS One. 2020 Dec 22;15(12):e0242881. doi: 10.1371/journal.pone.0242881 (PMC7755213; doi:10.1371/journal.pone.0242881)
Supplement: S1 Table — (DOCX) [file pone.0242881.s001.docx]

**S1 Table. HEART Item Descriptive Statistics: number of observations (n), mean, standard deviation (SD), median, minimum (min), maximum (max), skewness, kurtosis, and percentage of responses in response categories 1 through 6.**

| Item | n | Mean | SD | Median | Min | Max | Skew | Kurtosis | RC1 | RC2 | RC3 | RC4 | RC5 | RC6 |
| --- | --- | --- | --- | --- | --- | --- | --- | --- | --- | --- | --- | --- | --- | --- |
| Factor 1: Traditional Values | | | | | | | | | | | | | | |
| 1 | 308 | 3.02 | 2.12 | 2 | 1 | 6 | 0.39 | -1.61 | 42% | 13% | 5% | 6% | 10% | 25% |
| 2 | 308 | 2.6 | 1.93 | 1 | 1 | 6 | 0.7 | -1.15 | 51% | 10% | 5% | 11% | 9% | 14% |
| 3 | 308 | 1.93 | 1.72 | 1 | 1 | 6 | 1.58 | 0.84 | 73% | 5% | 5% | 3% | 5% | 10% |
| 4 | 308 | 2.42 | 1.66 | 2 | 1 | 6 | 0.81 | -0.69 | 47% | 14% | 13% | 11% | 9% | 6% |
| 5 | 308 | 2.79 | 1.88 | 2 | 1 | 6 | 0.54 | -1.28 | 39% | 19% | 5% | 11% | 12% | 13% |
| 6 | 306 | 1.74 | 1.47 | 1 | 1 | 6 | 1.95 | 2.48 | 74% | 7% | 6% | 4% | 3% | 7% |
| 7 | 308 | 1.95 | 1.61 | 1 | 1 | 6 | 1.51 | 0.82 | 66% | 10% | 6% | 4% | 7% | 7% |
| 8 | 308 | 2.63 | 1.81 | 2 | 1 | 6 | 0.64 | -1.1 | 45% | 13% | 7% | 14% | 10% | 11% |
| 9 | 308 | 2.67 | 1.76 | 2 | 1 | 6 | 0.55 | -1.21 | 41% | 16% | 7% | 13% | 15% | 7% |
| 10 | 308 | 2.59 | 1.93 | 1 | 1 | 6 | 0.69 | -1.17 | 52% | 9% | 5% | 10% | 10% | 14% |
| 11 | 308 | 2.82 | 2.05 | 2 | 1 | 6 | 0.49 | -1.48 | 48% | 8% | 5% | 9% | 11% | 19% |
| 12 | 307 | 2.3 | 1.68 | 1 | 1 | 6 | 1.04 | -0.29 | 50% | 16% | 11% | 7% | 8% | 8% |
| 13 | 307 | 2.28 | 1.86 | 1 | 1 | 6 | 1.07 | -0.51 | 61% | 8% | 5% | 6% | 7% | 12% |
| Factor 2: Partner Support | | | | | | | | | | | | | | |
| 1 | 305 | 4.66 | 1.76 | 5 | 1 | 6 | -1.14 | -0.15 | 13% | 4% | 4% | 9% | 22% | 48% |
| 2 | 308 | 5.31 | 1.27 | 6 | 1 | 6 | -2.13 | 3.78 | 4% | 4% | 2% | 6% | 19% | 66% |
| 3 | 308 | 4.17 | 1.96 | 5 | 1 | 6 | -0.44 | -1.44 | 15% | 13% | 12% | 6% | 8% | 45% |
| 4 | 307 | 3.74 | 1.93 | 4 | 1 | 6 | -0.13 | -1.51 | 19% | 14% | 14% | 11% | 11% | 31% |
| 5 | 307 | 4.94 | 1.73 | 6 | 1 | 6 | -1.36 | 0.28 | 10% | 6% | 5% | 6% | 8% | 66% |
| 6 | 307 | 3.44 | 1.85 | 3 | 1 | 6 | 0.14 | -1.39 | 21% | 14% | 23% | 8% | 10% | 24% |
| 7 | 308 | 4.56 | 1.83 | 6 | 1 | 6 | -0.9 | -0.71 | 13% | 6% | 9% | 10% | 10% | 53% |
| 8 | 307 | 5.13 | 1.5 | 6 | 1 | 6 | -1.76 | 1.83 | 7% | 4% | 3% | 6% | 17% | 64% |
| 9 | 308 | 5.2 | 1.59 | 6 | 1 | 6 | -1.83 | 1.81 | 8% | 4% | 4% | 3% | 7% | 74% |
| 10 | 306 | 5.06 | 1.34 | 6 | 1 | 6 | -1.56 | 1.75 | 4% | 3% | 6% | 12% | 22% | 54% |
| Factor 3: Partner Abuse & Control | | | | | | | | | | | | | | |
| 1 | 308 | 1.78 | 1.66 | 1 | 1 | 6 | 1.86 | 1.76 | 79% | 3% | 2% | 3% | 3% | 10% |
| 2 | 308 | 1.42 | 1.2 | 1 | 1 | 6 | 2.9 | 7.2 | 86% | 3% | 2% | 2% | 3% | 4% |
| 3 | 308 | 1.69 | 1.36 | 1 | 1 | 6 | 1.99 | 2.9 | 73% | 8% | 6% | 6% | 2% | 5% |
| 4 | 308 | 1.65 | 1.42 | 1 | 1 | 6 | 2.14 | 3.2 | 78% | 6% | 4% | 3% | 4% | 6% |
| 5 | 277 | 2.66 | 1.87 | 2 | 1 | 6 | 0.6 | -1.22 | 47% | 11% | 6% | 13% | 12% | 12% |
| 6 | 308 | 2.61 | 1.86 | 2 | 1 | 6 | 0.68 | -1.1 | 47% | 12% | 8% | 11% | 10% | 12% |
| 7 | 308 | 2.59 | 2.01 | 1 | 1 | 6 | 0.72 | -1.2 | 56% | 6% | 6% | 8% | 8% | 17% |
| 8 | 308 | 2.79 | 1.86 | 2 | 1 | 6 | 0.45 | -1.36 | 43% | 11% | 6% | 16% | 13% | 11% |
| 9 | 308 | 2.41 | 1.76 | 1 | 1 | 6 | 0.82 | -0.92 | 51% | 15% | 5% | 8% | 15% | 6% |
| Factor 4: Partner Resistance | | | | | | | | | | | | | | |
| 1 | 308 | 2.19 | 1.85 | 1 | 1 | 6 | 1.22 | -0.18 | 63% | 9% | 4% | 5% | 6% | 13% |
| 2 | 308 | 2.48 | 1.99 | 1 | 1 | 6 | 0.85 | -1 | 57% | 9% | 3% | 7% | 7% | 16% |
| 3 | 308 | 1.96 | 1.7 | 1 | 1 | 6 | 1.53 | 0.73 | 69% | 9% | 2% | 5% | 5% | 10% |
| 4 | 307 | 1.92 | 1.59 | 1 | 1 | 6 | 1.58 | 1.03 | 67% | 10% | 5% | 4% | 6% | 7% |
| 5 | 306 | 1.92 | 1.55 | 1 | 1 | 6 | 1.52 | 0.96 | 67% | 8% | 8% | 7% | 5% | 6% |
| Factor 5: HIV Prevention Readiness | | | | | | | | | | | | | | |
| 1 | 305 | 5.48 | 1.06 | 6 | 1 | 6 | -2.61 | 7.16 | 3% | 1% | 1% | 8% | 16% | 71% |
| 2 | 308 | 5.74 | 0.78 | 6 | 1 | 6 | -4.25 | 20.11 | 1% | 1% | 2% | 11% | 85% | 1% |
| 3 | 308 | 5.48 | 1.07 | 6 | 1 | 6 | -2.52 | 6.19 | 2% | 2% | 3% | 5% | 16% | 73% |
| 4 | 307 | 4.91 | 1.6 | 6 | 1 | 6 | -1.45 | 0.83 | 9% | 2% | 6% | 8% | 20% | 55% |
| 5 | 308 | 5.72 | 0.86 | 6 | 1 | 6 | -3.94 | 16.45 | 2% | 1% | 1% | 3% | 9% | 85% |
