# Supplementary material for: Development and initial validation of a simple tool to screen for partner support or opposition to HIV prevention product use
Source: PLoS One. 2020 Dec 22;15(12):e0242881. doi: 10.1371/journal.pone.0242881 (PMC7755213; doi:10.1371/journal.pone.0242881)
Supplement: S2 Table — (DOCX) [file pone.0242881.s002.docx]

**S2 Table. Illustrative quotes from IDIs used to predict HEART score ranges for Traditional Values, Partner Support, and Partner Resistance to HIV Prevention scales.**

| **ID** | **Score Prediction** | **Traditional Values Example Quotes** | **Rationale** |
| --- | --- | --- | --- |
| 1009 | Low | PARTICIPANT: Obviously each and every decision that I decide to take I must also tell him, and not decide alone. Even the same to him. Whatever decision that he’s deciding to take he must also tell me. I prefer it that way. | Participant and her partner are each able to make decisions and communicate them to each other, rather than her partner having the final say. |
| 1006 | Medium | PARTICIPANT: We know that men are in charge, we are unequal to them. When they speak, we must listen to them because that’s what we were taught when we were growing up. My mom listens to my father when he speaks.  INTERVIEWER: So what is happening in your relationship? PARTICIPANT: No, I don’t listen, if you want me to listen to you, you must listen to me also, we must understand each other you mustn’t control me. | Participant indicates that she is aware of "traditional values" in relationships from her parents but does not wish to follow them in her own relationship. |
| 1023 | High | INTERVIEWEE: Okay, it was about a week...we were not having sex for about a week because I was on my periods, and then he said I can’t take it anymore, can you make a plan, I just want to come. And I said, okay fine. Lets go and get condoms. We went and bought condoms and then we had sex. That was the only time he really convinced me to have sex, and I felt like if I don’t maybe he’s gonna cheat or he’s gonna call somebody else to sleep with him. Let me just give it to him. | Participant felt unable to refuse sex with partner, otherwise she may have to accept him cheating with other women. |
| **ID** | **Score Prediction** | **Partner Support Example Quote** | **Rationale** |
| 1026 | High | PARTICIPANT: Well he knows that I’ve being participating on ASPIRE. He was supportive about it actually, he was supportive because he is one person who doesn’t take anything that is health related simple, so ja he was actually supportive. | Partner was very supportive of participant using an HIV prevention product. |
| 1023 | Medium | INTERVIEWEE: ... We will fight but we don’t shout. We really have big fights, but we never shout at each other. We just talk and make sure that we resolve them. | Participant experiences big arguments with her partner, but they work together to resolve them. |

| 1012 | Low | PARTICIPANT: Like sometimes when I tell him like I want to go somewhere, he tells me like you are not going there. No, I want to go there. No, you are not, and would start a fight with that thing. Which is wrong. Or maybe if I say, maybe I’m going to visit my daddy. No, you’re not going there. Why? You are lying... Oh, okay. And I will never go because he said no, and I will not go, so I will stay. When I go somewhere, where are you going? Things like that, you know. You have to report everything, each and every move that you do. If you don’t do that he gets angry. | Participant experiences high tension in relationship and does not feel she can voice her own desires. |  |
| --- | --- | --- | --- | --- |
| **ID** | **Score Prediction** | **Partner Resistance to HIV Prevention Example Quote** | **Rationale** | |
| 1007 | Low | INTERVIEWER: The day you told him about your study participation, what was going on on that day that you told him? INTERVIEWEE: Actually, he was not stressed. He was happy. So I decided, yo, no I’m having something that I want to tell him that was really important. | Participant's partner was happy, not angry, to hear about her participation in ASPIRE. |  |
| 1032 | Medium | PARTICIPANT: I did. I can say I involved him. Because I was, even I was, I told him everything, there was nothing that I did in secret. I told him everything, but he was more negative about it. As I said he mentioned the gel study. He said they used people as experiments and left them like that. | Participant's partner was not very supportive of her joining ASPIRE due to what he had heard about other studies. However, she still told him about the study and he did not stop her participation. |  |
| 1011 | High | PARTICIPANT: I don’t really know what was going on in his mind, but at first when I told him he didn’t like the idea using a ring. ... He actually felt offended just because of he thought maybe I didn’t trust him. Maybe I’m using the ring because maybe I’m also sleeping with other guys, so I fear I’m gonna catch the virus so that is why I’m using the ring just to protect myself from being infected. | Participant's partner thought that use of an HIV prevention product might lead to her cheating on him. |  |
