# Supplementary material for: Development and initial validation of a simple tool to screen for partner support or opposition to HIV prevention product use
Source: PLoS One. 2020 Dec 22;15(12):e0242881. doi: 10.1371/journal.pone.0242881 (PMC7755213; doi:10.1371/journal.pone.0242881)
Supplement: S3 Table — (DOCX) [file pone.0242881.s003.docx]

| **ID** | **Low Score References (%)** | **Medium Score References (%)** | **High Score References (%)** | **Overall Score -  Prediction** | **Overall Score -  Actual** | **Correct?** |
| --- | --- | --- | --- | --- | --- | --- |

| **TRADITIONAL VALUES PREDICTIONS** | | | | | | |
| --- | --- | --- | --- | --- | --- | --- |
| 1006 | 26 | 26 | 49 | Medium | Medium | Yes |
| 1007 | 82 | 6 | 12 | Low | Low | Yes |
| 1009 | 56 | 44 | 0 | Low | Low | Yes |
| **1011** | 0 | 0 | 100 | High | Medium | No |
| 1012 | 0 | 60 | 40 | Medium | Medium | Yes |
| 1018 | 0 | 54 | 46 | Medium | Medium | Yes |
| 1019 | 18 | 49 | 33 | Medium | Medium | Yes |
| **1023** | 0 | 48 | 52 | High | Medium | No |
| 1026 | 10 | 65 | 25 | Medium | Medium | Yes |
| 1032 | 0 | 87 | 13 | Medium | Medium | Yes |
| **PARTNER SUPPORT PREDICTIONS** | | | | | | |
| 1006 | 36 | 44 | 20 | Medium | Medium | Yes |
| 1007 | 0 | 17 | 83 | High | High | Yes |
| 1009 | 24 | 70 | 6 | High | High | Yes |
| **1011** | 0 | 33 | 67 | High | Medium | No |
| 1012 | 65 | 35 | 0 | Low | Low | Yes |
| 1018 | 38 | 62 | 0 | Medium | Medium | Yes |
| **1019** | 5 | 67 | 28 | Medium | Low | No |
| 1023 | 0 | 55 | 45 | Medium | Medium | Yes |
| 1026 | 0 | 42 | 58 | High | High | Yes |
| 1032 | 8 | 65 | 27 | Medium | Medium | Yes |
| **PARTNER RESISTANCE TO HIV PREVENTION** | | | | | | |
| 1006 | 6 | 63 | 31 | Medium | Medium | Yes |
| 1007 | 52 | 48 | 0 | Low | Low | Yes |
| **1009** | 0 | 100 | 0 | Medium | Low | No |
| **1011** | 31 | 56 | 13 | Medium | Low | No |
| **1012** | 100 | 0 | 0 | Low | Medium | No |
| 1018 | 80 | 0 | 20 | Low | Low | Yes |
| 1019 | 9 | 69 | 22 | Medium | Medium | Yes |
| 1023 | 100 | 0 | 0 | Low | Low | Yes |
| 1026 | 66 | 34 | 0 | Low | Low | Yes |
| 1032 | 0 | 100 | 0 | Medium | Medium | Yes |
