# Supplementary material for: Development and initial validation of a simple tool to screen for partner support or opposition to HIV prevention product use
Source: PLoS One. 2020 Dec 22;15(12):e0242881. doi: 10.1371/journal.pone.0242881 (PMC7755213; doi:10.1371/journal.pone.0242881)
Supplement: S4 Table — (DOCX) [file pone.0242881.s004.docx]

**S4 Table. Examples of specific scale items reflected in qualitative interviews for Traditional Values, Partner Support and Partner Resistance to HIV Prevention.**

| **Item** | | | **# of Transcripts** | | **Traditional Values Example Quote** |
| --- | --- | --- | --- | --- | --- |
| 1. Changing diapers, giving the kids a bath, and feeding the kids is a mother's responsibility. | | | 3 | | I: It's normal for partners to not always agree on things. What types of things cause disagreements between you and your partner?  R: We disagree on ways we raise the children. Sometimes he makes me feel like I am wrong when I discipline the children |
| 2. I think that a woman cannot refuse to have sex with her husband. | | | 8 | | INTERVIEWER: Can you describe a time when you felt like your partner tried to convince you to have sex when you didn’t want? PARTICIPANT: I always want to, I don’t ever tell him when I don’t feel like doing sex. When he wants it I give him. |
| 3. I think that if a man has paid lobola for his wife, he owns her. | | | 0 | | N/A |
| 4. A woman should always listen and abide by the word of her husband without questions. | | | 8 | | INTERVIEWEE: Actually he feels every woman must be treated like human because every woman must talk for herself. Every woman for herself. If you don’t like something, you must talk, speak out because if you keep quiet how would he know that I’m not happy about it or not happy. He know I do not like that. He will not even know. So he feels like everyone must be equal. |
| 5. A man should have the final word about decisions in his home. | | | 8 | | I: Financial decisions and control can be another difficult thing to negotiate with a partner. How do you and your partner deal with finances? R: We try…with whatever that we have we sit down and try to decide on what we do. but still, I think now the problem is… most of the time, I do not feel he should make decisions about my money, but as a man I feel that he should be doing things for the house. |
| 6. A real man produces a male child. | | | 0 | | N/A |
| 7. I think that if a man has paid lobola for his wife, she must have sex when he wants it. | | | 0 | | N/A |
| 8. I think that a man should have the final say in all family matters. | | | 8 | | INTERVIEWER: How would decisions be made about what you discuss and do?  INTERVIEWEE: Obviously each and every decision that I decide to take I must also tell him, and not decide alone. Even the same to him. Whatever decision that he’s deciding to take he must also tell me. I prefer it that way. |
| 9. A woman should accept her partner's wishes – even when she disagrees - to keep the family together. | | | 8 | | I: Okay. What does it mean for women not to be equal to men?  R: Women must respect their men, because some men have anger and then they don’t want a lady respond to them or maybe he is fighting with you. Then you answer back in anger, you see, he will end up beating you up without that intention. That is why I can say that. |
| 10. I only think I am attractive if other people think I am. | | | 1 | | INTERVIEWER: How does it make you feel when his family’s saying such things about you? INTERVIEWEE: It makes me feel like I’m his princess. He doesn’t make me feel like I’m old. Because what I know, he always tells me you’re smart, you always try to keep yourself looking good, even though you’re not working. I know I might not be giving you much but you’re always up to date. He always make me feel like I’m beautiful. No matter what people say. |
| 11. I think that there is nothing a woman can do if her husband wants to have girlfriends. | | | 4 | | INTERVIEWER: Can you tell me about the times when your partner tried to convince you to have sex with him when you didn’t want to?INTERVIEWEE: Okay, it was about a week...we were not having sex for about a week because I was on my periods, and then he said I can’t take it anymore, can you make a plan, I just want to come. And I said, okay fine. Lets go and get condoms. We went and bought condoms and then we had sex. That was the only time he really convinced me to have sex, and I felt like if I don’t maybe he’s gonna cheat or he’s gonna call somebody else to sleep with him. Let me just give it to him. |
| 12. If someone insults a man, he should defend his status with force if he has to. | | | 0 | | N/A |
| 13. It's only rape if a woman fights back. | | | 2 | | INTERVIEWEE: When I say “Ey, today I’m tired.” He’ll be like” Yah, how can you be tired? It means there’s someone you were sleeping with.” “Like what are you trying to say?” “Yah, I’m telling you. You are cheating at me Celesta. If you can tell me that you’re tired and you only sleep with me, it means there’s someone else you’re sleeping with.” I’ll say “There’s no one I’m sleeping with.” He’ll say “If there’s no one then just open your legs.” Like, do you understand? So it was like a forced sex which it was like rape. |
| **Item** | | | **# of Transcripts** | **Partner Support Example Quote** | |
| 1. My partner is as committed as I am to our relationship. | | | 8 | INTERVIEWEE: I feel like a queen. I get everything that I want, even [indistinct], I talk to him. He’s very supportive. INTERVIEWER: When you say you get everything that you want, like? INTERVIEWEE: Support, maybe I’m in need of something, I need to go out and stuff, do my hair. Like now I’m not working. He’s very supportive, still. | |
| 2. I feel comfortable telling my partner that I see things differently. | | | 10 | PARTICIPANT: No, I don’t listen, if you want me to listen to you, you must listen to me also, we must understand each other you mustn’t control me. | |
| 3. In general, my relationship has a lot of tension. | | | 7 | R: Because we don't, we discuss about everything and we don't fight a lot, and I think we do compromise. | |
| 4. It takes a long time to work out arguments with my partner. | | | 3 | INTERVIEWEE: No. Me if I see that now he’s angry I shut my mouth, or go out to my friends or to my neighbours. If its me who is angry, he don’t talk with me. He sleep. So, that’s the way we handle our situation. | |
| 5. I feel trapped or stuck in our relationship. | | | 3 | I: I know. But sometimes its hard because we try to avoid fighting and then it just happens. Did it ever happen where you kind of just got into a fight? R: A lot of times ... a lot of times. Because I felt like I was just tired of him controlling me, telling me what to do, telling me what not to do. Like, he was in control of my life and I wasn’t really free. So I fought back. | |
| 6. Arguments with my partner result in me feeling down or bad about myself. | | | 1 | INTERVIEWEE: Everything is well. We do fight but using those [indistinct] after that we can’t be sad. We can’t be sad for a long time. | |
| 7. My partner does what he wants, even if I do not want him to. | | | 6 | INTERVIEWEE: Even now I still don’t know. Hey. I want to know him, but I’m failing. I’m failing to know him, because he can’t tell you want he want, what he don’t want. He just do sometimes actions, sometimes, ah. | |
| 8. I feel safe in my current relationship. | | | 4 | INTERVIEWEE: I’m scared, maybe...okay, maybe I’m scared, okay I put the trust in him, what if maybe he hurts me, what if...what if he’s playing me. I never know. What if he’s pretending. Something like that. | |
| 9. My partner takes my earning or refuses to give me money when he has money for other things. | | | 3 | PARTICIPANT: We trust each other but when it comes to finances we don’t trust each other because he taught me to hide my money from him because that’s what he is doing to me. | |
| 10. My partner is/will be very supportive of my use of an HIV prevention product. | | | 7 | INTERVIEWEE: It’s the same partner. I had one partner. He was very supportive, because he used to remind me of the dates. When are you going to the clinic? He even used to come with me, or even if I came by myself, he would come and fetch me. | |
| **Item** | | **# of Transcripts** | | | **Partner Resistance to HIV Prevention Example Quote** |
| 1. If I asked my partner to use a condom, he would get angry. | | 3 | | | INTERVIEWEE: Ja. But what he didn’t want is to use condom. Yo. He would say your results was fine, it was negative, so why I using condom. You have a ring. You want me to use a condom again? No. So even if you try to explain to him, ah, he can’t even see. |
| 2. If I asked my partner to use a condom, he would think I'm having sex with other people. | | 4 | | | INTERVIEWEE: We did talk about it. Because I was like “why, why don’t you like using condoms? What is it about it? He’ll say “ the condom hurt me.” |
| 3. If I asked my partner to use a condom, he would get violent. | | 0 | | | N/A |
| 4. I cannot tell my partner about HIV prevention product use because he will become angry. | | 5 | | | R: I don’t really know what was going on in his mind, but at first when I told him he didn’t like the idea using a ring. He didn’t understand why, even though I tried explaining to him that I’m using this ring for this and that. |
| 5. If I asked my partner to use an HIV prevention product, he would get violent. | | 0 | | | N/A |
| **Item** | **# of Transcripts** | | | | **HIV Prevention Readiness Example Quote** |
| 1. I understand the risks and benefits of HIV prevention product use and have chosen to use them. | 7 | | | | INTERVIEWEE: Because it was a very wrong thing, like how will it enter inside my vagina. I was told how to use it and how to put it. So after a while I was very comfortable with it. Because I was scared what if I got my periods what would happen to the ring. Nothing happened. They explained to me you won’t feel anything. You just have to keep it until your dates. |
| 2. Using an HIV prevention product is the right thing to do. | 6 | | | | INTERVIEWEE: When they explained the study to me they said it was a prevention. They’re trying to find a cure, a prevention for women because most of the time women, most of the ladies they cannot say no if the partner is saying you must not use protection, stuff like that. So they want to see if this would work, so that’s what made me to join the study. |
| 3. Using an HIV prevention product shows that my partner and I care about each other. | 4 | | | | INTERVIEWEE: It was important because...what can I say...he was going to benefit too from the study. |
| 4. Using an HIV prevention product with my partner will help us communicate better. | 5 | | | | PARTICIPANT: It made me closer to him because he knew about it and he understood. He didn’t have a problem, he knew that I inserted it. He got used to it and didn’t feel that it was there. |
| 5. HIV prevention products would help me protect myself. | 10 | | | | R: Ja. Because despite of everything he was doing, I felt like I should also protect myself from getting infected by him because he wasn’t faithful to me at all. So for myself it was just go to participating in Aspire, it was for my own good and safety. |
